# Contribution of pelvic and para-aortic lymphadenectomy with sentinel node biopsy in patients with IB2–IIB cervical cancer

**DOI:** 10.1038/bjc.2011.541

**Published:** 2011-12-06

**Authors:** E Chéreau, J-G Feron, M Ballester, C Coutant, C Bezu, R Rouzier, E Touboul, E Daraï

**Affiliations:** 1 Department of Gynecology-Obstetrics, Hôpital Tenon, Assistance Publique des Hôpitaux de Paris, CancerEst, Université Pierre et Marie Curie Paris 6, 4 rue de la Chine, Paris 75020, France; 2 Department of Radiotherapy, Hôpital Tenon, Assistance Publique des Hôpitaux de Paris, CancerEst, Université Pierre et Marie Curie Paris 6, Paris 75020, France

**Keywords:** pelvic lymphadenectomy, paraaortic lymphadenectomy, locally advanced cervical cancer, sentinel node biopsy, survival

## Abstract

**Objective:**

Detection of lymph node involvement in women with IB2–IIB cervical cancer could have a positive effect on survival. We set out to evaluate the incidence of pelvic and/or para-aortic lymph node involvement using the sentinel node (SN) biopsy and its impact on survival.

**Methods:**

From 2002 to 2010, 66 women with IB2–IIB cervical cancer underwent a pelvic and paraaortic lymphadenectomy with SN biopsy. Survival between groups according to lymph node status was evaluated.

**Results:**

Mean tumour size was 43.5 mm. At least one SN was detected in 69% of the 45 SN procedures performed. Sixteen of these patients had metastatic SN and the false negative rate was 20%. Metastatic pelvic SNs or non-SNs were detected in 33 patients (50%), including pelvic-positive nodes in 26 (40%), pelvic- and paraaortic-positive lymph nodes in seven (11%), and paraaortic skip metastases in two (6%). Positive paraaortic node was the sole determinant for disease-free survival (DFS) and overall survival (OS; *P*<0.001). Differences in DFS and OS between groups according to the nodal status were observed (*P*<0.001).

**Conclusion:**

SN procedure gave a higher rate of metastasis detection. Further studies are required to evaluate whether pre-therapeutic node staging, including paraaortic and pelvic lymphanedectomy, should be performed.

Despite a recent revision of the FIGO (International Federation of Gynecology and Obstetrics) classification, cervical cancer continues to be the only gynaecological malignancy that is not surgically staged (Petru *et al*, 2009). This contributes to difficulties in evaluating the effect of therapy, particularly of lymphadenectomy, on survival for locally advanced stages of cervical cancer (higher than or equal to stage IB2). Indeed, lymph node involvement is relatively frequent in locally advanced stages of cervical cancer and is a major determinant for adjuvant therapy (Zander *et al*, 1981; Shepherd, 1996; Morice and Castaigne, 2005).

Imaging techniques including CT, MRI and PET have a high diagnostic accuracy for evaluating enlarged lymph nodes, but a poor accuracy for regular-sized lymph nodes (Hricak *et al*, 1988; Kim *et al*, 1993, 1994; Subak *et al*, 1995; Boss *et al*, 2000; Sheu *et al*, 2001; Hertel *et al*, 2002; Narayan *et al*, 2003; Kamelle *et al*, 2004; Marnitz *et al*, 2005; Selman *et al*, 2008). Systematic lymphadenectomy is thus recommended to evaluate metastases in pelvic and/or para-aortic lymph nodes (PALNs) (Zander *et al*, 1981; Piver, 1984; Lanciano *et al*, 1991; Chu *et al*, 1997; Michel *et al*, 1998; Stryker and Mortel, 2000; Vergote *et al*, 2002; Narayan *et al*, 2003). Sentinel node (SN) biopsy has also become widespread to determine lymph node status in early stages of cervical cancer (Selman *et al*, 2008; Altgassen *et al*, 2009). However, the interest of the SN biopsy in locally advanced stages of cervical cancer is more debatable, because of the low SN detection rate and high false negatives (Barranger *et al*, 2003, 2004a, 2004b; Coutant *et al*, 2007; Altgassen *et al*, 2009).

Although a prospective study has shown a survival disadvantage for patients following surgical staging compared with clinical staging when concurrent radiochemotherapy (CRC) is recommended (Lai *et al*, 2003), most authors agree that lymph node status should be assessed by systematic lymphadenectomy. However, a debate exists whether paraaortic lymphadenectomy alone is sufficient or whether a pelvic and paraaortic lymphadenectomy should be performed systematically. Leblanc *et al* (2007) recommended a paraaortic lymphadenectomy alone, considering that CRC possibly associated with localised boost on positive pelvic nodes and/or on the parametria are sufficient to control local regional disease. In contrast, Houvenaeghel *et al* (2006) demonstrated persistence of active pelvic lymph node metastases after CRC, and that pelvic lymphadenectomy could reduce the rate of lateropelvic recurrences whatever the PALN status.

Therefore, the aim of the present retrospective study was to evaluate the incidence of pelvic and/or PALN involvement, using both SN biopsy and systematic pelvic and paraaortic lymphadenectomy, and the impact on survival in women with advanced stages of cervical cancer (stage IB2 or II).

## Patients and methods

### Patients

From 2002 to 2010, 66 women with locally advanced cervical cancer corresponding to 1988 FIGO stage IB2 or II underwent a pre-therapeutic pelvic and paraaortic lymphadenectomy by laparoscopy in the gynaecology unit of Tenon Hospital, France (Barranger *et al*, 2003, 2004a, 2004b; Coutant *et al*, 2007). All the women had biopsy-proven cervical cancer and had undergone pelvic MRI, and 45 of the 66 women had undergone a laparoscopic SN procedure before pelvic and paraaortic lymphadenectomy.

All women gave informed written consent to the therapeutic procedures and to the analysis of data related to their malignancy in accordance with institutional guidelines and the Declaration of Helsinki. The protocol was approved by the local Ethics Committee.

The medical records were reviewed to determine age, the body mass index, tumour stage, histology, tumour size on MRI, surgical procedure, intra- and postoperative complications, and the final pelvic and paraaortic node status. Outcome was obtained from the outpatient records.

The predictive factors for disease-free survival (DFS) and overall survival (OS) were analysed in univariate and multivariate analysis to provide survival data. Survival between groups according to their nodal histological status – positive or negative pelvic nodes and positive or negative paraaortic nodes – was evaluated.

### Technique

#### SN procedure

Ths SN procedure was performed as previously reported (Barranger *et al*, 2003; Coutant *et al*, 2007). The pelvic and lower paraaortic regions were carefully inspected by laparoscopy for lymph ducts and dye uptake by lymph nodes. All blue and/or hot lymph nodes were removed separately. The position of each SN relative to the major pelvic vessels, vena cava or aorta was recorded.

After the SN procedure, systematic transperitoneal lymph node dissection extending from the external iliac (and obturator nerve) to the level of the left renal vein was performed. The absence of residual pelvic or paraaortic radioactivity was verified before and after pelvic and paraaortic lymphadenectomy.

Lymph nodes with macroscopic metastases were sectioned. Normal-appearing SNs were cut perpendicular to the long axis. All SNs were submitted to intra-operative imprint cytology. Air-dried cytological smears were prepared by scraping the cut surfaces and staining with a rapid May-Grünwald–Giemsa method. Each half-SN was sectioned at 3-mm intervals. Each 3-mm section was analysed at four additional levels of 150 *μ*m and four parallel sections; one was used for hematoxylin and eosin (H&E) staining, and H&E-negative sections were examined by immunohistochemistry (IHC) with an anticytokeratin antibody cocktail (cytokeratins AE1-AE3; Dako Corporation, Glostrup, Denmark). Non-SNs were submitted totally and blocked individually after 3-mm sectioning and H&E staining.

Macrometastases was defined by a single focus of metastatic disease per node measuring more than 2 mm, micrometastases as a focus of metastatic disease ranging from 0.2 mm to no more than 2 mm and, in accordance with previous studies (Marchiole *et al*, 2005; Bezu *et al*, 2010), submicrometastases as metastases measuring no more than 0.2 mm including the presence of a single non-cohesive tumour cell. SNs and non-SNs were considered positive when they contained macrometastases, micrometastases or submicrometastases.

### Concurrent radiochemotherapy

External pelvic radiation therapy was given through four orthogonal fields: antero-posterior (AP) and postero-anterior (PA), and two lateral fields. The upper limit of the AP/PA field was L4–L5 interspace. The lower limit extended distally to the midportion of the obturator foramen or the lowest level of disease with a 3-cm margin, and laterally 2 cm beyond the lateral margins of the bony pelvic wall. The upper and lower limits of the lateral fields were the same as those of the AP/PA field. The anterior limit of the lateral field was a horizontal line drawn at the anterior border of the pubic symphysis. The posterior limit of lateral field was placed at the S2–S3 interspace. Customised blocks were used to spare the anterior half of the rectum posteriorly and a proportion of small bowel anteriorly.

Pelvic radiation therapy consisted of 40 Gy, using 2.25 Gy per fraction, 4 days a week. A vaginal booster dose of 20 Gy was given at 5–6 weeks by means of brachytherapy. Brachytherapy was performed after radical hysterectomy when uterine catherisation was impossible.

Concurrent chemotherapy was given during the 1st and 4th week of radiation therapy and consisted of a continuous 5-fluorouracil infusion (750 mg m^−2^ per day) and a cisplatin bolus (20–25 mg m^−2^ per day) 1 h before radiotherapy, for days 1, 2, 4 and 5. When the pelvic and paraaortic nodes were not involved, simple or radical laparoscopic hysterectomy was performed 6 weeks after the end of CRC. Women with positive lymph node involvement underwent a specific CRC regimen. For these women, the total dose of external radiotherapy delivered was 45 Gy with an iliac boost of 10 Gy, followed by the same brachytherapy regimen. The chemotherapy protocol was the same, but delivered the 1st and the 5th week of irradiation.

Patients with positive aortic nodes received extended-field radiation up to the level of T12-L1. The lateral limits were set 4 cm from the midline.

### Statistical analysis

Data were analysed using the *χ*^2^-test or the Fisher's exact test and the Student's *t*-test. Differences were considered significant when *P*<0.05. OS time was calculated in months from the date of surgery to death, or the date of last follow-up for surviving patients and DFS time from the date of surgery to recurrence. The Kaplan–Meier method was used to estimate the survival distribution, and comparisons of survival were made by the use of the log-rank test. Cox proportional hazards regression was used for multivariate analysis. Informative prognostic factors for outcome were selected according to Akaike Information Criteria.

## Results

### Epidemiological and surgical characteristics of the population

Patient and tumour characteristics are reported in Table 1. The mean tumour size was 43.5 mm (range: 12–70). Eighty-six percent of the patients had a squamous cell carcinoma. About half of the patients had a moderately or poorly differentiated carcinoma, and more than half of the patients had FIGO stage IIB.

All 66 patients underwent both pelvic and paraaortic lymphadenectomy for a cervical cancer. This procedure was followed by CRC and brachytherapy for 58 of them (88%). A total of 2 of the 66 patients required a conversion to laparotomy: for anaesthesiological disorders related to hypoventilation for one and for ureteral injury requiring a bladder reimplantation for the other. No bleeding or vascular injury requiring laparotomy was observed. Moreover, none of these patients had unresectable bulky nodes. Eight patients (12%) with stage II A cervical cancer and tumour size below 4 cm underwent a laparoscopic pelvic and paraaortic lymphadenectomy associated with a radical hysterectomy during the same surgical procedure, followed by CRC and brachytherapy.

### SN procedure

SN procedure was performed in 45 patients, resulting in the detection of at least one SN in 69% of cases (*n*=31; Table 2). A bilateral SN was found in 26% of cases (*n*=12). The mean number of SN removed was 2.1 (1–4) per patient. No difference in cervical cancer stages between patients with and without SN was detected.

Among these 45 patients, in 87% of cases (39 among 45 cases), SN was hot and blue, in 5 cases, SN was blue alone (11%) and in 1 case, SN was radioactive alone (2%).

Histology revealed metastatic SN in 16 patients (52%, 16 among 31 patients with at least one SN detected), including macrometastases in 12 cases and micrometastases in 4 (25%). All positive SN were distributed in the pelvic area and had positive HES staining. Four patients had a false negative SN giving a false negative rate of 20%.

### Pelvic and paraaortic lymphadenectomy

The mean number of lymph nodes removed, including SNs during pelvic or paraaortic lymphadenectomy, were 12.5 nodes (3–24) and 12.5 nodes (4–28), respectively. Metastatic pelvic SNs or non-SNs were detected in 33 patients (50%). Metastatic PALNs were detected in nine patients (14%). Skip metastases to PALNs were diagnosed in two patients in the presence of negative pelvic lymph nodes. In these two patients, the first presented 1-mm micrometastases with one negative SN identified (HES and IHC). The second patient did not have a SN biopsy and presented 10 pelvic nodes free from disease and one of the 28 PALNs involved.

Among the 35 patients (53%) with positive nodes, 20% of them had both pelvic and paraaortic positives nodes, 74% had only pelvic positives nodes and 6% had isolated paraaortic positives nodes. In patients with isolated pelvic positive nodes, 83% of them had both metastatic SNs and non-SNs, whereas 17% had only metastatic SNs. None had only metastatic non-SNs.

Four false negative cases of SN procedure were observed corresponding to patients with unilateral SN detection. In three of them, only one SN was removed and in the last case, two SNs were removed. The four patients also had positive PALNs.

### OS and DFS

**Figure 1:**
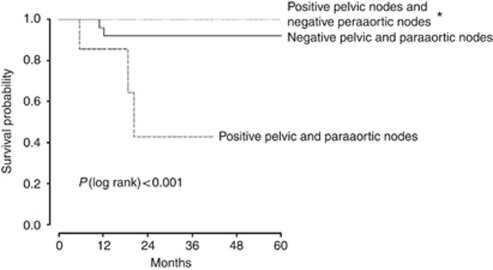
Disease-free survival according to nodal status in 66 patients with stage Ib2–IIb cervical cancer. ^*^No significant difference in survival between patients with negative pelvic and paraaortic nodes and patients with positive pelvic nodes and negative paraaortic nodes.

**Figure 2:**
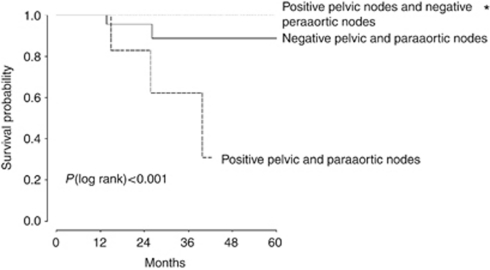
Overall survival according to nodal status in 66 patients with stage Ib2–IIb cervical cancer. ^*^No significant difference in survival between patients with negative pelvic and paraaortic nodes and patients with positive pelvic nodes and negative paraaortic nodes patients.

The median follow-up was 28.3 months (2–79 months; Figures 1 and 2). Seven of the sixty-six patients (11%) relapsed, including three centropelvic recurrences (two of three in patients without hysterectomy), two peritoneal carcinomatosis (11 and 17 months after surgery), one lateropelvic and one common iliac node recurrences. The 5-year DFS was 86% and the 5-year OS was 78%. Univariable analysis for DFS found that the only significant factor was the positivity of paraaortic nodes (*P*<0.001). This factor was also significant in multivariable analysis (*P*=0.02). For OS, positive paraaortic nodes (*P*<0.0001) remained the sole determinant factor in univariable, but not in multivariable analysis (Table 3).

The OS and PFS of the patients were assessed in three groups: no lymphatic disease, only pelvic-positive nodes and both pelvic- and paraaortic-positives nodes. For both DFS and OS, we found statistical differences between these groups with a *P*-value <0.001 (Figures 1 and 2).

## Discussion

The present study demonstrates the high incidence of pelvic and PALN involvement and the contribution of the SN biopsy to detect micrometastases in patients with locally advanced stages of cervical cancer.

A recent meta-analysis (Chemoradiotherapy for Cervical Cancer Meta-analysis Collaboration (CCCMAC), 2010)) including 13 trials has confirmed that the gold standard to treat patients with locally advanced-stages cervical cancer is CRC: a 6% improvement in 5-year survival with chemoradiotherapy (hazard ratio=0.81, *P*<0.001) was observed when chemoradiotherapy was compared with the same radiotherapy. However, this meta-analysis based on lymph node status, especially iliac node involvement, was not completed as there were insufficient data not allowing to state on the impact of lymphadenectomy. We observed a high incidence of pelvic and PALN involvement, 50% and 14%, respectively, in our study. However, despite a significant difference in both OS and PFS between patients with positive pelvic and paraaortic positive nodes, and patients with only positive pelvic nodes, we cannot conclude that lymphadenectomy has a therapeutic effect, as no difference was observed between patients with and without positive pelvic lymph nodes. This explains why some authors recommend paraaortic lymphadenectomy only (preferentially by retroperitoneal approach) to determine the extent of radiotherapy while limiting the side effects on small bowel, suggesting that adjuvant chemoradiation is able to sterilise all pelvic lymph nodes in patients with locally advanced-stages cervical cancer. However, Houvenaeghel *et al* (2006) found that 16% of women with locally advanced cervical cancer initially treated by CRC had persistent positive pelvic lymph nodes. In a series of 73 patients with IB2–IIB cervical cancer treated by CRC, followed by paraaortic lymphadenectomy associated with pelvic lymphadenectomy or pelvic lymph node sampling in 36 patients, Morice *et al* (2007) reported that 13 patients (36%) had persistent positive pelvic lymph node after CRC. In the study of Morice *et al* (2007), among the four pelvic lymph node relapses, three occurred in patients who had not undergone pelvic lymphadenectomy. Moreover, in a multivariate analysis, Rouzier *et al* (2005) demonstrated that, in addition to tumour size, the main determinant of pelvic relapse was pelvic lymph node involvement. Therefore, in addition to prognostic relevance, pelvic lymphadenectomy may have a therapeutic impact by reducing the risk of lymph node relapse, thus reinforcing the idea that when lymphadenectomy is indicated before CRC, both pelvic and paraaortic lymphadenectomy should be performed. Indeed, Marnitz *et al* (2005) showed that removal of positive pelvic and/or positive PALNs was associated with significant improvement in OS, confirming that lymphadenectomy should be performed before primary chemoradiation. Comparing survival of patients undergoing negative PALN identified by surgical staging to patients with only radiographic exclusion of PALN metastases, Gold *et al* (2008) showed that patients with radiographic evaluation only had a poorer prognosis supporting the therapeutic effect of lymphadenectomy. Moreover, Tseng *et al* (2010) built a nomogram in patients with locally advanced-stage cervical cancer, showing a high heterogeneity in predicting death, but underlined the preponderant impact of both pelvic and paraaortic involvement. Finally, our results underline that three-quarters of patients with lymph node metastases were located in the pelvis, whereas only 20% had pelvic and paraaortic involvement, and only 6% had isolated paraaortic metastases. These data are of particular relevance, as two trials included in a recent meta-analysis (Lukka *et al*, 2002; Green *et al*, 2005; Chemoradiotherapy for Cervical Cancer Meta-analysis Collaboration (CCCMAC), 2010) showed greater benefits of adding chemotherapy after CRC, with an absolute improvement of 19% at 5 years. Patients with lymph node involvement, especially with pelvic and/or paraaortic metastases, could be good candidates for this new regimen.

Our SN detection is low compared with those observed in patients with early stages of cervical cancer (Plante *et al*, 2003; Martinez-Palones *et al*, 2004; Coutant *et al*, 2007), but concur with those of previous studies on SN in locally advanced stages of cervical cancer (Coutant *et al*, 2007). This difference in detection rates may be explained by the obstruction of lymphatic vessels by tumour embols. Moreover, we found a high false negative rate of 20% in the present study, contrasting with that of Altgassen *et al* (2009) reporting a false negative rate under 10% for tumour size below 2 cm for early stages of cervical cancer. All these considerations underline that SN procedure cannot be considered an alternative to lymphadenectomy in patients with locally advanced stages of cervical cancer. Despite a low SN detection and a high false negative rate, our results underline the contribution of ultrastaging, using combined serial sectioning and IHC to detect micrometastases; 20% of our patients with lymph node involvement were exclusively diagnosed as a result of ultrastaging. Lentz *et al* (2004), using IHC without serial sectioning, detected micrometastases in 19 out of a series of 132 women with 3106 negative lymph nodes on routine histology (15% 95% confidence interval: 9–22%). Silva *et al* (2005) confirmed the contribution of IHC in detecting micrometastases in 5 of 98 negative SNs. In a recent review on SN biopsy in cervical cancer, using H&E and IHC (Lambaudie *et al*, 2003; Martinez-Palones *et al*, 2004; Niikura *et al*, 2004; Kraft *et al*, 2006) on SNs, no micrometastases were detected. Using H&E, serial sectioning and IHC, the incidence of micrometastases ranged from 0% to 47.4% with a mean value of 28.3%, similar to that observed in the current study.

From a clinical view point, Juretzka *et al* (2004) first underlined the potential prognostic relevance of micrometastases and recommended adjuvant therapy for these patients. In a case–control study, Marchiole *et al* (2005) found that the relative risk of recurrence in the presence of true micrometastases (focus of metastatic disease ranging from 0.2 mm to no more than 2 mm) was 2.30 (confidence interval: 1.65–3.20, *P*<0.01). Moreover, in series of 894 patients, Horn *et al* (2008) confirmed the prognostic relevance of detecting micrometastases with a correlation between their presence and the risk of recurrence. Hence, all these data reinforce the notion that patients with metastases, including those with micrometastases detected in SNs, could be candidates for adjuvant chemotherapy after CRC.

Some limitations of the present study have to be underlined. First, the retrospective nature of the study cannot exclude the risk of potential bias. Second, no difference was observed between patients with and without positive pelvic lymph nodes, raising the issue on the rational of systematic pelvic lymphadenectomy. However, this could be explained by both the sample size of the study and the relatively short follow-up with few events (seven recurrences). Concerning the rational for completion of the surgery in our protocol, it is clear that no consensus exists on its indication and on its impact on survival while exposing patients to the risk of potential severe postoperative complications. Third, despite the contribution of ultrastaging using combined serial sectioning and IHC to detect micrometastases, our study was unable to prove the therapeutic effect of pelvic lymphadenectomy. This could suggest that pre-therapeutic pelvic lymphadenectomy is unnecessary, as pelvic radiotherapy could be sufficiently effective on positive pelvic nodes. However, even in new regimens of radiotherapy or chemoradiation, there is a lack of data on pelvic node sterilisation, particularly, when using radiotherapy boost (Haie-Meder *et al*, 2009).

## Conclusion

The SN procedure resulted in an increased detection rate of pelvic node metastases, which are often underestimated, despite a high false negative rate. According to a recent meta-analysis showing the benefits of adding chemotherapy after CRC in case of lymph node metastases, patients with lymph node metastases could be good candidates for this regimen. Further studies are required to evaluate whether pre-therapeutic node staging, including paraaortic and pelvic lymphanedectomy, should be performed in women with locally advanced cervical cancer.

## Figures and Tables

**Table 1:** Epidemiological and surgical characteristics of the 66 patients with locally advanced stages of cervical cancers, who underwent pelvic and para-aortic lymphadenectomy and sentinel lymph node biopsy

**Characteristics**	**Patients (*n*=66)**
Mean age in years (range)	48.8 (28–76)
Post-menopausal patients (%)	26 (39)
Mean body mass index, kg m^−2^ (range)	23.4 (16.8–35.0)
Mean tumour size on MRI, mm (range)	43.5 (12–70)
	
*Tumour location in the cervix*	
Ectocervical (%)	62 (94)
Endocervical (%)	3 (5)
Exo and endocervical (%)	1 (1)
	
*Tumour histology*	
Squamous cell carcinoma (%)	57 (86)
Adenocarcinoma (%)	9 (14)
	
*Histological grade of the tumour*	
Well differentiated (%)	30 (45)
Moderately differentiated (%)	10 (15)
Poorly differentiated (%)	15 (23)
Unclassified (%)	11 (17)
	
*FIGO classification*	
IB2 (%)	23 (34)
IIA (%)	8 (12)
IIB (%)	35 (54)
	
*Therapy associated with LPPAL*	
Chemoradiotherapy and brachytherapy (%)	27 (41)
Chemoradiotherapy and brachytherapy, followed by hysterectomy (%)	31 (47)
First radical hysterectomy and LPPAL, followed by chemoradiotherapy and brachytherapy (%)	8 (12)

Abbreviations: FIGO=International Federation of Gynecology and Obstetrics; LPPAL=laparoscopic pelvic and para-aortic lymphadenectyomy; MRI=magnetic resonance imaging.

**Table 2:** Results of the SN biopsy and of pelvic and para-aortic lymphadenectomy in 66 patients with stage IB2–IIB cervical cancer

**Characteristics**	**Number of patients (%)**
*Pelvic SN*	
Number of patients (%)	45 (68)
At least one identified SN (%)	31 (69)
Mean number of SNs per patient (range)	2.1 (1–4)
Patients with bilateral SN (%)	12 (26)
	
*Histopathology (among 31 patients with SN detected)*	
Negative SN (%)	15 (48)
Positive SN (%)	16 (52)
Macrometastasis (%)	12 (39)
Micrometastasis (%)	4 (13)
	
*Pelvic non-SN*	
Mean number of LNs per patient (range)	12.5 (3–24)
	
*Pelvic SN and non-SN*	
Number of patients	66
Number of patients with metastatic LN (%)	33 (50)
SN performed (%)	20 (60)
SN+/non-SN+ (%)	7 (21)
SN+/non-SN− (%)	9 (27)
SN-/non-SN+ (%)	4 (12)
False negative rate (%)	4/20 (20)
SN detection not performed (%)	13 (40)
	
*Para-aortic lymph node*	
Number of patients	66
Mean number of LNs per patient (range)	12.5 (4–28)
Number of patients with metastases (%)	9 (14)
	
*Patients with positive nodes*	
Number of patients (%)	35 (53)
Positive pelvic and paraaortic nodes (%)	7 (20)
Positive isolated pelvic nodes (%)	26 (74)
SN+/non-SN+ (%)	19 (83)
SN+/non-SN− (%)	7 (17)
SN-/non-SN+	0
Positive isolated para-aortic nodes (%)	2 (6)
SN performed	1
Pelvic negative SN detected	1

Abbreviations: LN=lymph node; SN=sentinel node.

**Table 3:** Univariable and multivariable analysis of potential predictive factors of pelvic or para-aortic lymph node metastasis in 42 patients with stage IB2–IIB cervical cancer

	**Pelvic lymph node metastases**	**Para-aortic lymph node metastases**
	**Univariable analysis (*P*)**	**Univariable analysis (*P*)**
Tumour size >30 mm	0.81	1
Tumour size >40 mm	0.38	0.88
FIGO stage	0.01	0.43
Post-menopausal status	0.44	0.3
Age	0.12	0.88
Histology	0.15	1

Abbreviation: FIGO=International Federation of Gynecology and Obstetrics
